# A retrospective study comparing interventions by oncology and non‐oncology pharmacists in outpatient chemotherapy

**DOI:** 10.1002/cnr2.1371

**Published:** 2021-03-19

**Authors:** Michihiro Kaya, Kazuyo Nakamura, Makiko Nagamine, Yukako Suyama, Michiaki Nakajo, Ryo Uchida, Kakeru Hagikura, Ai Kanda, Kyohei Sugiyama, Rina Sugiyama, Shigeru Nakagaki, Midori Kimura

**Affiliations:** ^1^ Department of Pharmacy Shizuoka General Hospital Shizuoka City Japan

**Keywords:** chemotherapy, clinical pharmacy services, interventions, pharmacist, retrospective study

## Abstract

**Background:**

The differences in the clinical pharmacy services (CPS) provided by oncology and non‐oncology pharmacists have not been sufficiently explained.

**Aim:**

This study aimed to demonstrate the differences in direct CPS provided by oncology and non‐oncology pharmacists for patients and physicians, and to assess the potential impact of these services on medical costs.

**Methods:**

We retrospectively examined CPS provided by oncology and non‐oncology pharmacists for outpatients who underwent chemotherapy between January and December 2016.

**Results:**

In total, 1177 and 1050 CPS provided by oncology and non‐oncology pharmacists, respectively, were investigated. The rates of interventions performed by oncology and non‐oncology pharmacists for physicians‐determined treatment were 18.5% and 11.3%, respectively (*p* < .001). The rates of oncology and non‐oncology pharmacist interventions accepted by physicians were 84.6 and 78.8%, respectively (*p* = .12). Level 4 and Level 5 interventions accounted for 64.6% of all oncology pharmacist interventions and 53.0% of all non‐oncology pharmacist interventions (*p* = .03). The rates of improvement in symptoms from adverse drug reactions among patients resulting from interventions by oncology and non‐oncology pharmacists were 89.4 and 72.1%, respectively (*p* = .02). Conservative assessments of medical cost impact showed that a single intervention by an oncology and by a non‐oncology pharmacist saved ¥6355 and ¥3604, respectively.

**Conclusion:**

The results of the present study suggested that CPS by oncology pharmacists enable safer and more effective therapy for patients with cancer and indirectly contribute to reducing health care fees.

## 1 INTRODUCTION

Novel antineoplastic agents are being developed in large numbers, thus increasing options for therapy. At the same time, this proliferation of antineoplastic agents brings about a more complicated process in the use of chemotherapy, and heightens the importance of measures for adverse drug reactions (ADRs) other than hematotoxicity. Consequently, pharmacists engaged in cancer therapy, are expected to serve many other roles.^1^, ^2^, ^3^ Many studies have reported on the contributions of pharmacists to cancer therapy, including many Japanese studies.^4^, ^5^, ^6^, ^7^ Due to the high level of expert knowledge and skills required to fulfill these roles, Japan has created a system to train and certify oncology pharmacists (OP). For a pharmacist to be qualified for OP, the following items must be implemented. (a) Receive 5 years of training on cancer and cancer chemotherapy according to the program created by the society. (b) Submit 50 cases of clinical pharmacy services (CPS) in which pharmacists performed excellent interventions to patients undergoing cancer chemotherapy to the academic society. (c) Conduct continuous research activities on cancer. (d) Pass the OP certification examination. In addition, the OP must renew his/her qualification every 5 years. Several critical studies have reported on the services performed by OP.^8^, ^9^ One major Japanese study reported that the participation by OP in drug therapy can reduce medical costs and make therapy safer.^10^ Another recent study has shown that intervention by pharmacists resulted in more suitable pharmacotherapy.^11^ However, to the best of our knowledge, no study has ever compared the CPS conducted by OP with that conducted by non‐oncology pharmacists (non‐OP). Therefore, we aimed to demonstrate the differences between direct CPS provided by OP and non‐OP for patients and physicians and to assess the potential impact of these services on medical costs.

## 2 MATERIALS AND METHODS

### 2.1 Intravenous chemotherapy practice at Shizuoka General Hospital

The present study is a retrospective comparison of services provided by pharmacists for outpatients undergoing chemotherapy at Shizuoka General Hospital, which is designated by the Japanese Ministry of Health, Labour, and Welfare as a Regional Core Hospital. This hospital provides high‐quality medical care and plays a primary role in cancer care in central Shizuoka Prefecture. The Chemotherapy Center has 40 beds and provides outpatient chemotherapy on weekdays. To administer intravenous chemotherapy at this hospital, a physician must select a regimen that is previously registered in the electronic medical records. These regimens are based on evidence such as cancer therapy guidelines and clinical trials. Once a physician selects a regimen, the doses of the antineoplastic agents are automatically calculated based on the individual patient's background characteristics. Also registered with these regimens are the recommended doses, routes, and rates of administration of infusions and supportive therapy injections necessary to administer the neoplastic agents. Therefore, by selecting the necessary regimen, physicians can provide consistent therapy. However, the physician can adjust the dosage based on the patient's condition. Before receiving antineoplastic agents, the patient is examined by a physician; if deemed eligible, the patient visits the Chemotherapy Center to commence chemotherapy.

At the Chemotherapy Center, CPS, which the management of ADRs and education for individual patients are conducted on weekdays by one OP and one non‐OP. CPS for physicians includes pharmaceutical intervention; for example, if any problems arise in therapy, the pharmacist along with the physician intervenes to improve therapy. In Japan, pharmacists are not authorized to make the final decision regarding the instructions such as the selection of pharmacotherapy or the necessary tests. Therefore, in principle, pharmacists require a physician's permission to conduct pharmaceutical interventions for patients, to improve their therapy. Pharmacists always perform CPS when patients are administered with a given antineoplastic agent for the first time; these services may be continued if necessary. The present study was conducted in accordance with the Declaration of Helsinki and other good clinical practice guidelines with the approval of the Shizuoka General Hospital Institutional Review Board (approval no.: SGHIRB#2017043).

### 2.2 Study design

We retrospectively compared CPS provided by OP and non‐OP for patients who underwent outpatient intravenous chemotherapy. All CPS were abstracted from the medical records and all endpoints were evaluated from the CPS with the type of pharmacist blinded. After evaluation, CPS were classified as either OP or non‐OP service, and compared.

### 2.3 Endpoints

The primary endpoint is the rate of pharmaceutical interventions conducted by pharmacists with the physicians. The secondary endpoints are: (a) the rate of the physicians' acceptance of interventions, (b) quality of interventions, (c) rate of improvement in ADRs, and (d) medical cost impact per CPS based on the avoidance of ADR exacerbation and prevention of ADRs.

The intervention rate was calculated as the number of patients for which pharmacists intervened with physicians divided by the total number of CPS. The physician acceptance rate was calculated as the number of interventions accepted by the physicians and conducted by the pharmacists divided by the total number of interventions. Quality of interventions was assessed as follows. The rate of interventions was classified on a five‐level scale from the highest to the lowest as follows: Level 5) intervention requiring advanced knowledge and response; Level 4) intervention in accordance with the oncology guidelines or package inserts; Level 3) intervention in response to the general ADRs or changes in symptoms; Level 2) simple interventions such as changes in the prescription duration or prescription errors by the physicians; and Level 1) incorrect interventions due to misjudgment by the pharmacist. This scale is the original scale and was set up in this study to assess the quality of interventions. Intervention levels were classified by a co‐author who was not the principal author. The rate of Level 4 or 5 interventions was then reclassified. For ADR improvement rate, we compared the rates of improvement in patients' symptoms following the physician's acceptance of intervention, among all interventions for ADRs.

Calculations of the economic impact of the pharmaceutical interventions were based on a study by Tasaka et al^12^ In Japan, the Pharmaceuticals and Medical Devices Agency (PMDA) has a relief system in place for patients who develop serious ADRs. In 2016, the PMDA paid out a total of ¥2 267 542 000 for 1340 incidences of serious ADRs (a mean of approximately ¥1 692 537 per case) to victims or their surviving relatives. Based on this data, we estimated the medical cost impact of avoiding a serious ADR or exacerbation of an ADR to be approximately ¥1 692 000 per case (2 267 542 000 ÷ 1340). According to several studies, the mean continuous pharmaceutical intervention rates by pharmacists that led to the prevention of major ADRs or of exacerbation of ADRs was 5.21%.^13^, ^14^, ^15^ Hamblin et al. believed that therapy using a Web‐based system halved the risk of ADRs and also assessed the interventions by pharmacists.^16^ Based on the above, we also believed that 2.6% of individual pharmaceutical interventions accepted by physicians led to the prevention of major ADRs or of exacerbation of ADRs; we divided the product of this figure and the estimated per‐case PMDA payment by the total number of CPS to estimate the medical cost impact per service.

### 2.4 Statistical analysis

The sample size was calculated based on the number of CPS at Shizuoka General Hospital and past studies. The numbers of CPS for outpatients conducted by OP and non‐OP at Shizuoka General Hospital are similar. Sudou et al. reported that the rate of interventions by pharmacists before the examination by a physician is 17.6%.^17^ Based on this figure, we predicted a mean baseline pharmaceutical intervention rate of 15.0% and hypothesized that the rate for OP would be 5% higher than the rate for non‐OP. Using a two‐sided test (*α* = .05, *β* = .10), we calculated that we would need to analyze a total of 2220 CPS. We began registering cases in January 2016 and continued until the month when the number of registered cases exceeded 2220. As a result, we analyzed CPS provided between January and December 2016. Because this was a retrospective study, it was difficult to obtain informed consent directly. Therefore, the study was conducted with a public document stating the study outline and the option for patient refusal to participate in the study. Percentages were compared using Fisher's exact test. All statistical analyses were performed with EZR (Saitama Medical Center, Jichi Medical University, Saitama, Japan), which is a graphical user interface for R (the R Foundation for Statistical Computing, Vienna, Austria). More precisely, it is a modified version of R commander, designed to add the statistical functions frequently used in biostatistics.^18^ The differences were considered statistically significant at *p* < .05.

## 3 RESULTS

### 3.1 Patients characteristics

Patient characteristics is shown in Table 1. During the study period, a total of 2227 CPS were performed by five OP (1177 services) and six non‐OP (1050 services). Neither group showed any evident imbalance in patient characteristics.

**TABLE 1:** Patient characteristics^a^

Characteristic	OP (*n* = 1177)	Non‐OP (*n* = 1050)
Chemotherapy‐naïve patients	433 (36.8)	389 (37.0)
Median age (range), yrs	61 (23‐91)	63 (25‐90)
Sex		
Male	442 (37.6)	421 (40.1)
Female	735 (62.4)	629 (59.9)
Medical Department		
Breast surgery	400 (34.0)	267 (25.4)
Medical Oncology	346 (29.4)	266 (25.3)
Obstetrics and Gynecology	195 (16.6)	198 (18.9)
Respiratory Medicine	141 (12.0)	178 (17.0)
Gastroenterology	65 (5.5)	80 (7.6)
Gastroenterological Surgery	10 (0.8)	36 (3.4)
Urology	8 (0.7)	17 (1.6)
Hematology	9 (0.8)	5 (0.5)
Dermatology	2 (0.2)	2 (0.2)
Otorhinolaryngology, Head and Neck Surgery	1 (0.1)	1 (0.1)

Abbreviation: OP, oncology pharmacist.

^a^ Data are given as a number (percentage) unless otherwise indicated.

### 3.2 Clinical pharmacy services

Specific CPS are shown in Table 2. During the study period, OP conducted a total of 266 interventions for 218 patients, while non‐OP conducted a total of 132 interventions for 119 patients. Significantly higher intervention occurred with OP (18.5%) than with non‐OP (11.3%; *p* < .001). The intervention rate for patients receiving a given neoplastic agent for the first time was significantly higher among OP than among non‐OP (OP vs. non‐OP: 14.5 vs. 8.7%, *p* = .01). The intervention rate for all other patients was also significantly higher among OP than among non‐OP (OP vs. non‐OP: 20.8 vs. 12.9%, *p* < .001).

**TABLE 2:** Clinical pharmacy services and interventions^a^

	OP	non‐OP	*p* Value
Pharmacists	5	6	
Clinical pharmacy services	1177	1050	
Patients intervened for	218	119	
Interventions	266	132	
Acceptances	225	103	
			
Overall intervention rate	218 (18.5)	119 (11.3)	<.001
First administration intervention rate (%)	63 (14.5)	34 (8.7)	.01
Other (%)	155 (20.8)	85 (12.9)	<.001
			
Total physician acceptances	225	103	
Total acceptance rate	225 (84.6)	103 (78.0)	.12
Chemotherapy intervention acceptance rate	74 (78.7)	20 (69.0)	.32
Supportive therapy intervention acceptance rate (%)	34 (91.9)	25 (75.8)	.10
ADR intervention acceptance rate(%)	117 (86.7)	58 (82.9)	.53
			
Intervention quality			
Rate of Level 4–5 interventions (%)	64.6	53.0	.03
Level 5 (%)	15 (5.6)	2 (1.5)	
Level 4 (%)	157 (59.0)	68 (51.5)	
Level 3 (%)	60 (22.6)	28 (21.2)	
Level 2 (%)	34 (12.8)	33 (25.0)	
Level 1 (%)	0 (0)	1 (0.8)	
			
ADR interventions accepted by physicians	85	43	
Improvement (%)	76 (89.4)	31 (72.1)	.02
No change (%)	8 (9.4)	9 (20.9)	
Exacerbation (%)	1 (1.2)	3 (7.0)	
ADR intervention targets			
Leukopenia/neutropenia	7	1	
Anemia	4	0	
Thrombocytopenia	1	1	
Electrolyte imbalance	6	2	
Nausea/vomiting	24	14	
Constipation/diarrhea	9	6	
Stomatitis	4	1	
Peripheral neuropathy	4	0	
Allergic reaction	2	1	
Skin disorder	7	5	
Abnormal blood pressure	3	2	
Liver disease	2	0	
Kidney disease	3	0	
Angialgia	2	1	
Other	7	9	

Abbreviations: ADR, adverse drug reaction; OP, oncology pharmacist.

^a^ Data are given as a number (percentage) unless otherwise indicated.

Rates of interventions accepted by physicians did not differ significantly between OP (84.6%) and non‐OP (78.0%; *p* = .12). Interventions were divided into three categories for comparison: interventions associated with antineoplastic agents, interventions associated with other agents, and interventions to improve ADRs that had occurred in patients. In all three categories, the rate of acceptance by physicians was higher among OP; however none of the differences were significant for OP versus non‐OP, respectively (antineoplastic agents, 78.7 vs. 69.0%, *p* = .32; supportive care agents, 91.9 vs. 75.8%, *p* = .10; ADRs, 86.7 vs. 82.9%, *p* = .53).

We subsequently compared the quality of OP vs non‐OP interventions which were based on Level 4‐5 intervention rates. Significantly higher rates were found among OP than among non‐OP, and these were 64.6% (Level 5, 5.6%; Level 4, 59.0%) and 53.0% (Level 5, 1.5%; Level 4, 51.5%), respectively (*p* = .03).

We then compared OP and non‐OP in terms of symptom improvement rates where interventions for improving ADRs were accepted by the physician, and thereafter, the patient's symptoms were assessed. For both OP and non‐OP, the most common ADR targeted for intervention was nausea/vomiting, followed by constipation/diarrhea. A significantly higher rate of ADR improvement was found among OP than among non‐OP which was 89.4 and 72.1%, respectively (*p* = .02).

### 3.3 Estimated medical cost impact

Table 3 shows the formula and data for estimating the medical cost impact as well as the medical cost impact per CPS. ADR improvement interventions accepted by physicians were higher among OP than among non‐OP which was 14.4 and 8.2%, respectively (*p* < .001). The medical cost impact per CPS was then calculated as described in section 2.3 and according to the formula shown in Table 3 and was then estimated to be ¥6355 for OP and ¥3604 for non‐OP.

**TABLE 3:** Estimated medical cost impact

Formula Medical cost impact = Pharmaceutical interventions accepted by physicians *a*) × 2.6% × Mean Adverse Drug Reaction Relief System payment per case *b*)/Clinical pharmacy services			
	OP	Non‐OP	*p* Value
Clinical pharmacy services	1177	1050	
ADR improvement interventions accepted by physicians *a*)	170 (14.4%)	86 (8.2%)	< .001
Mean Adverse Drug Reaction Relief System payment per case *b*) (JPY)	1 692 000	1 692 000	
Medical cost impact (JPY/CPS)	6355	3604	

*Note: a*) Individual pharmaceutical interventions accepted by physicians. Does not include interventions requiring additional orders for laboratory data required for the safety management of pharmaceuticals. *b*) 2016 PMDA data.

Abbreviations: ADR, adverse drug reaction; CPS, clinical pharmacy services; JPY, Japanese Yen; OP, oncology pharmacist; PMDA, Pharmaceuticals and Medical Devices Agency.

## 4 DISCUSSION

The present study is a highly novel retrospective study in which we assessed and directly compared a sufficient number of CPS provided by OP and non‐OP (Table 1). The study period included more CPS provided by OP than by non‐OP, but this difference likely did not have a major effect on our analysis. The intervention rate among OP was significantly higher than that among non‐OP. Our hospital provides chemotherapy according to a highly‐regulated regimen system. Therefore, incorrect administration routes, rates of administration, or ADR measures that deviate from the guidelines are uncommon at the commencement of therapy. Thus, the number of interventions addressing these issues was fairly low. From the results in Table 2, the rate of Level 4‐5 interventions and improvement of ADR after performed by OP was significantly higher than that performed by the non‐OP, it is considered that OP has the higher skill to intervene in complex problems and better ADR management than non‐OP. In a study by Sudou et al., when CPS were performed before a patient was examined by a physician, the intervention rate was 17.6%.^17^ In the present study, all CPS were performed after examination by a physician. Although the study by Sudou et al. and the present study cannot be so easily compared, the fact that physicians try to optimize pharmacotherapy for individual patients by examining them suggests that rates of intervention by pharmacists would be lower when performed after examination than before examination. When this assumption is considered, the intervention rate among OP in the present study was sufficiently higher than the intervention rate reported by Sudou et al., indicating that CPS provided by OP is of extremely high quality. While the rate of interventions by non‐OP (11.3%) was lower than that found in the figure reported by Sudou et al., the line of reasoning described above suggests that non‐OP also sufficiently exercised their capabilities as pharmacists.

We cannot say from the present study whether the benefit to patients was greater when pharmacist interventions were accepted by a physician or not accepted. However, the high rates of physician acceptance of interventions by OP (84.6%) and non‐OP (78.0%) suggest that the pharmacists' interventions were fundamentally appropriate. We must consider why the rates of acceptance did not differ significantly between OP and non‐OP. Comparisons of intervention quality showed that the rate of Level 4‐5 consultations was significantly higher among OP than among non‐OP. Although the data is not shown, the mean rates of physician acceptance of pharmacist interventions (by OP and non‐OP) were 78.9% for Level 4‐5 interventions and 87.8% for Level 1‐3 interventions. Thus, proposals for Level 4‐5 interventions were accepted less frequently. This phenomenon may have relatively reduced the rate of acceptance of intervention proposals by OP in the present study.

The reasons for intervention by pharmacists for patients may vary widely but often include improving symptoms, preventing ADRs, and support for appropriate pharmacotherapy. Improvement in symptoms following physician acceptance is important in intervention for improving ADRs. Both groups of pharmacists had high rates of ADR improvement, indicating excellent intervention; however, intervention by OP was considered to have contributed even more to therapy. Our data on medical cost impact was also highly meaningful. It is extremely difficult to elucidate the incidences and details of ADRs and exacerbated ADRs that would have occurred without intervention by a pharmacist. Therefore, in Table 3, based on studies by Tasaka et al. and Hamblin et al., we calculated the medical cost impact of interventions by pharmacists. In the Japanese health care system, a fee of ¥2000 can be charged when an OP provides instruction to a patient undergoing chemotherapy. In the present study, the estimated medical cost impact of one OP intervention was estimated to be ¥6355 (Table 3), which suggests that the fee was not sufficiently high. Although we could not calculate the fee for instruction by non‐OP, their estimated medical cost impact of ¥3604 per intervention indicates a major effect of non‐OP interventions not only on ADR improvement but also on medical costs.

Our study had several limitations. Because this study was a retrospective study, we were unable to randomize patients into OP and non‐OP groups. Generally, interventions were performed by one pharmacist for each patient; however, patients could get advice from other pharmacists. To minimize bias, pharmacist records were blinded and three co‐authors identified a comparison of intervention quality and ADR improvement rates. We calculated the medical cost impact of interventions by pharmacists from multiple studies. Since the intervention by pharmacists would help mitigate ADRs, the cost of actual ADRs could only be predicted. For these reasons, intervention effect assessment should be viewed as hypothesis‐generating only. This study focused on outpatient chemotherapy; therefore, further studies are warranted to examine the effect of the interventions for hospitalized patients.

## 5 CONCLUSIONS

The present study demonstrated the differences in the CPS provided by OP and non‐OP and the impact of their interventions on medical costs. While CPS provided by both OP and non‐OP greatly contributed to cancer therapy and reduction in medical costs, CPS by OP was, particularly of higher quality. The impact of CPS by both OP and non‐OP may exceed the medical fees currently being charged for their services. Therefore, CPS for outpatients who undergo chemotherapy may not only provide better clinical management for patients but also reduce medical costs. Overall, the CPS performed by the OP has a greater effect than the CPS performed by the non‐OP. Therefore, nurturing OPs to support more treatments is also an important issue.

## AUTHOR CONTRIBUTIONS

**Michihiro Kaya:** Conceptualization; data curation; formal analysis; investigation; methodology; project administration; supervision; visualization; writing‐original draft; writing‐review & editing. **Kazuyo Nakamura:** Conceptualization; investigation; writing‐review & editing. **Makiko Nagamine:** Investigation; writing‐review & editing. **Yukako Suyama:** Investigation; writing‐review & editing. **Michiaki Nakajo:** Investigation; writing‐review & editing. **Ryo Uchida:** Investigation; writing‐review & editing. **Kakeru Hagikura:** Investigation; writing‐review & editing. **Ai Kanda:** Investigation; writing‐review & editing. **Kyohei Sugiyama:** Investigation; writing‐review & editing. **Rina Sugiyama:** Investigation; writing‐review & editing. **Shigeru Nakagaki:** Investigation; writing‐review & editing. **Midori Kimura:** Conceptualization; project administration; writing‐review & editing.

## CONFLICT OF INTEREST

The authors declare no conflict of interest.

## ETHICS STATEMENT

The Shizuoka General Hospital institutional review board approved this study. The ethical committee exempted the researchers from obtaining patient consent directly based on the retrospective and non‐interventional characteristics of the study (SGHIRB#2017043). Instead of obtaining patient consent directly, the study was conducted with a public document stating the study outline and the option for patient refusal to participate in the study.

## Data Availability

Data sharing is not applicable to this article as no new data were created or analyzed in this study.
